# The substernal implantable cardioverter-defibrillator: First experience combining an extravascular-implantable cardioverter-defibrillator with a dual-chamber transvenous pacemaker

**DOI:** 10.1016/j.hrcr.2023.11.004

**Published:** 2023-11-23

**Authors:** Hans Römers, Max Liebregts, Vincent van Dijk, Lucas Boersma

**Affiliations:** ∗ Department of Cardiology, Sint Antonius Hospital, Nieuwegein, The Netherlands; † Department of Cardiology, Amsterdam University Medical Centers, University of Amsterdam, Amsterdam, The Netherlands

**Keywords:** EV-ICD, Transvenous pacemaker, Oversensing, Ventricular tachycardia, Heart block

**Key Teaching Points**

• When combining 2 functional devices in 1 patient, always check for cross-talk in normal setting but also in the worst-case scenario; this is to avoid potential problems that might occur during the lifetime of the devices or leads. You need to be prepared for those possible issues.
• It is always possible that after implantation of a new type of device, a new indication occurs for that patient. Just explanting and implanting a new device that fits both indications is not always necessary. Be creative in a safe way and see what is already done in the literature for possible options, or consult expert centers for advice.
• Consult expert centers or company staff for advice. They might have already encountered such a problem and may be able to assist with troubleshooting.

## Introduction

The implantable cardioverter-defibrillator (ICD) is an accepted therapy for protection against life-threatening arrhythmias. However, the use of transvenous leads may have drawbacks, including bleeding, perforation, pneumothorax, thrombosis, and infection. The development of a subcutaneous ICD (S-ICD) system with a lead parallel to the left sternal border has obviated the transvenous lead. However, the subcutaneous position does not allow options for bradycardia or antitachycardia pacing (ATP).

The development of the extravascular ICD (EV-ICD)^1^^,^^2^ enables ATP by a substernal position of the lead on the heart. In the current version, only short-term back-up pacing is available for bradycardia, owing to the relatively high output settings required to stimulate the heart. In this case report we present a patient with an active EV-ICD implant that developed a need for a transvenous pacemaker (PM) for continuous pacing.

## Case report

**Figure 1:**
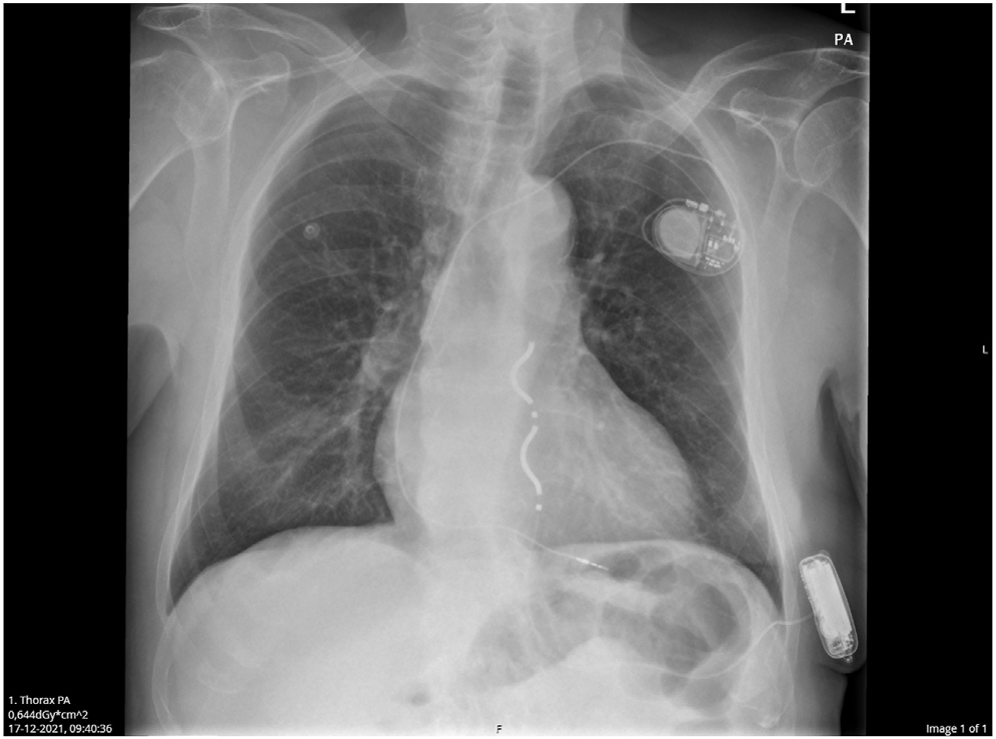
Radiography after device implant showing single-chamber pacemaker and extravascular implantable cardioverter-defibrillator.

A 71-year-old man with a severe concentric hypertrophic cardiomyopathy, essential hypertension, normal left ventricular ejection fraction (55%), and persistent atrial fibrillation experienced ventricular tachycardia (VT) with loss of consciousness that required electrical cardioversion. The electrocardiogram (ECG) at baseline showed atrial fibrillation (AF) with a left bundle branch block (LBBB) and QRS duration of 130 ms. Angiography did not reveal any coronary artery stenosis. An EV-ICD for secondary prevention was implanted in the beginning of 2021 as part of the IDE/CE trial. The EV lead was placed through a subxyphiodal incision and tunneled into the substernal space through a small hole in the diaphragm. Fluoroscopy confirmed correct position of the coils over the cardiac figure toward the right and electrodes toward the left side (Figure 1). The EV-ICD generator was placed in the left lateral position. Electrical parameters were tested and showed stable R waves ≥2 mV. Defibrillation threshold testing was performed twice successfully according to study protocol. In the first test ventricular fibrillation (VF) was induced, with good VF sensing and detection at a sensitivity level of 0.45 mV and successful defibrillation at 20 J. In the second test the first shock failed at 15 J but the second attempt at 30 J converted VF back to sinus rhythm. Max shock energy in the EV-ICD is 40 J and a safety margin of at least 10 J is maintained. The patient was discharged from the hospital the next day after ECG confirmed sinus rhythm, radiography confirmed proper system position, and device interrogation showed satisfactory electrical parameters and programming.

Two weeks after implantation the first device follow-up was performed. All measurements were within the normal expected range. ECG showed AF with heart rates of 70–80 beats per minute (bpm). One episode of nonsustained tachycardia was seen, but this was AF with rapid conduction without triggering ICD therapy.

**Figure 2:**
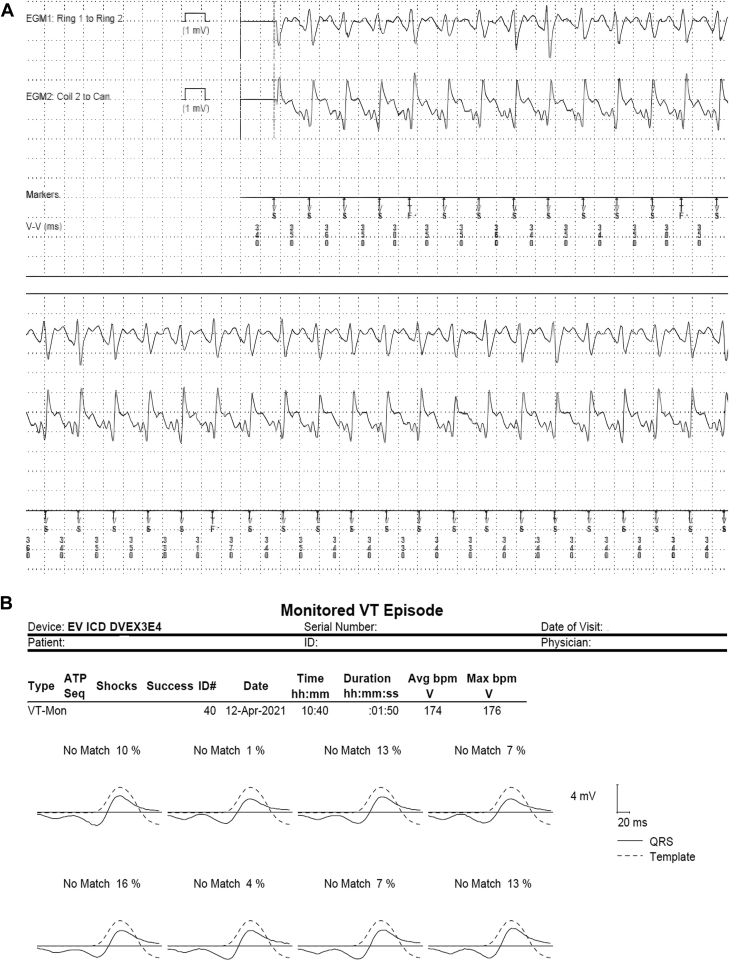
**A:** Ventricular tachycardia event. **B:** Morphology scoring.

At 2 months follow-up of the device again showed several AF episodes with rapid conduction. Discrimination of this arrhythmia was flawless, so no therapy was given by the device. In addition, 1 episode of nonsustained VT at 170 bpm was found in the monitor zone (Figure 2A). Wavelet correctly recognized this as VT (Figure 2B). Since the patient experienced dizziness during the VT event and had multiple dizzy spells in the past 2 month, the monitor zone was lowered to 150 bpm and active Fast VT zone was lowered to 162 bpm with therapies available.

**Figure 3:**
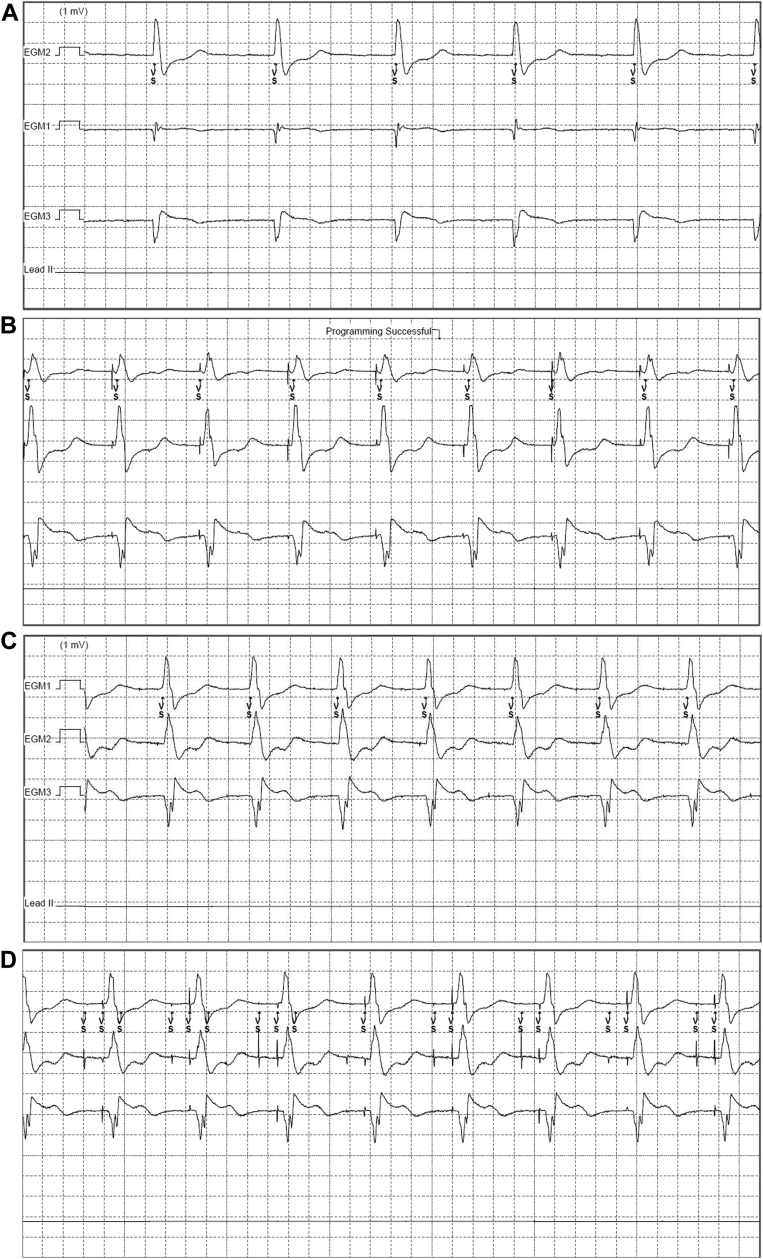
**A:** Electrogram (EGM) of the extravascular implantable cardioverter-defibrillator (EV-ICD) before pacemaker implant. **B:** EGM of the EV-ICD during right ventricular pacing. **C:** EGM of the EV-ICD with dual-chamber bipolar pacing in optimal sensitivity (0.150 mV) and Sensing Threshold Drop Time (1500 ms). **D:** EGM of EV-ICD with dual unipolar pacing (8 V and 1.5 ms) with EV-ICD at max sensitivity (0.075 mV) and shortest Sensing Threshold Drop Time (500 ms).

Six months later the patient presented at the emergency department with a loss of consciousness while cycling, without ICD therapy. Device interrogation showed no tachycardia or bradycardia events. The patient reported regular events of dizzy spells. The ECG now showed right bundle branch block instead of the previous LBBB. The patient was admitted and telemonitoring confirmed alternating right bundle branch block and LBBB with persistent atrial fibrillation. The decision was made to implant a single-chamber transvenous Medtronic Astra PM owing to the concern for high-grade atrioventricular (AV) block or infrahisian block as the most likely cause of the syncope in accordance with ESC guidelines for pacing.^3^ During follow-up both PM and EV-ICD performed as expected, with all values within normal range and no oversensing during pacing (Figure 3A and 3B) Some months later the patient returned to the outpatient clinic with signs of PM syndrome owing to spontaneous recurrence of sinus rhythm with bradycardia and AV synchrony. This necessitated upgrade to a Medtronic Astra dual-chamber PM. The EV-ICD did not register any oversensing of dual-chamber pacing, even at deliberate high-output pacing (Figure 3C). At last follow-up the patient showed a complete AV block with high percentage of both atrial (74%) and ventricular pacing (95%) without any sign of oversensing. There were no events recorded by the EV-ICD that would suggest that oversensing occurred since last device follow-up.

## Discussion

Implant of an EV-ICD has its advantages by avoiding venous access issues and possible complications of a transvenous lead, similar to an S-ICD. While the EV-ICD enables short bursts of ATP, bradycardia pacing options remain limited at this time. Concomitant implantation of a venous pacemaker system with the S-ICD is well described.^4^, ^5^, ^6^, ^7^, ^8^, ^9^ In this case, the patient developed syncope and alternating bundle branch block that prompted the need for pacing.

The clinical situation of the patient left us with 2 options: adding a separate pacemaker or implanting a transvenous ICD with full pacing options and explanting the EV-ICD. The EV-ICD has limited back-up pacing capability. Pacing only starts after a 5-second pause and uses a high-voltage output. Initial data^10^ indicate that substernal pacing is perceived as uncomfortable, probably owing to the high output necessary to stimulate the heart. These high pacing voltages would quickly lead to battery depletion. Therefore frequent pacing by the EV-ICD is undesirable. At the time of first PM implant, there was no evidence of AV block yet. The EV-ICD had not yet provided any back-up pacing. The treating physicians at that time decided to implant a back-up PM because of the alternating bundle branch block. Since the patient had been in chronic AF for as long as known, there was no need for a dual PM. A leadless PM might have been an alternative, but the treating physicians did not choose that option for practical reasons. Furthermore the physicians decided that it might be best to choose a conventional transvenous PM, given that the patient was part of an IDE study, and a leadless PM was considered a more experimental approach than a transvenous PM.

After implantation of a transvenous PM, testing for oversensing is necessary to avoid false detection of pacing artifacts in the EV-ICD. We tested the worst-case scenario for oversensing while pacing is at 8 V in unipolar configuration. The EV-ICD is set at lowest sensitivity setting of 0.075 mV and shortest Sensing Threshold Drop Time of 500 ms. The Sensing Threshold Drop Time algorithm prevents oversensing the p wave. A time delay withholds the algorithm for a certain time (500 ms) before it lowers its setting to its minimal sensitivity. Oversensing of atrial or ventricular pacing artifact is likely to happen (Figure 3D). Optimal threshold for pacing leads at implant is an important consideration in patients with existing EV-ICD to avoid oversensing with higher output in the future.

In this case optimal pacing parameters were set at 2.0 V output with 0.4 ms duration, bipolar lead configuration in the pacemaker, and in the EV-ICD sensitivity of 0.150 mV. A Sensing Threshold Drop Time of 1500 ms was programmed. This resulted in no oversensing events and correct sensing of the QRS by the EV-ICD.

### Limitations

The knowledge gained in this case report is not automatically transferable to every other patient, and an individual assessment will always have to be made.

## Conclusion

This case report highlights that the simultaneous implantation of a TV-PPM and an EV-ICD is safe and feasible, without any disruption to the functionality of either device. Initially, atrial (2.6%) and ventricular (4.1%) pacing demands were low, but they increased during follow-up. Remarkably, there were no oversensing issues with the EV-ICD despite this, suggesting that it would be well tolerated in the long term. Future application of our findings will depend on individual patient characteristics.
